# Morphometric Evaluation of Seminiferous Tubules in Aged Mice Testes after Melatonin Administration

**Published:** 2011-04-21

**Authors:** Fereshteh Mehraein, Feraidoon Negahdar

**Affiliations:** Anatomy Department, Medical School, Tehran University of Medical Sciences, Tehran, Iran

**Keywords:** Melatonin, Seminiferous Tubules, Morphology, Aged Mice

## Abstract

**Objective:**

Melatonin, the pineal gland hormone as a direct or indirect antioxidant and free radical scavenger, is involved in the process of both aging and age-related diseases. This study investigates the effects of melatonin on the histology of testicular seminiferous tubules in aged mice.

**Materials and Methods:**

Twenty male, white mice, aged 16 months, that weighed 20-23 gr were equally divided into control and experimental groups. The experimental group was intraperitoneally injected with a daily single dose of 10 mg/kg melatonin for 14 days. The control group received only saline. Six days after the last injection, all mice were sacrificed and the testes were excised and processed for light microscope observation. In the morphometric study, we evaluated testicular seminiferous tubule parameters such as height of germinal epithelium, seminiferous tubule diameter, thickness of interstitial connective tissue and spermatogenesis index (SI). SPSS software and student's t-test analyzed all parameters to assess the significance of changes between control and experimental groups.

**Results:**

Melatonin-treated mice had seminiferous tubules with a wide lumen lined by low height germinal epithelium. The interstitial connective tissue thickened significantly in the experimental group (p<0.05), tubular diameter and germinal epithelium height decreased significantly (p<0.01), and the SI reduced compared to the control group (p<0.001).

**Conclusion:**

The results of this study showed the disadvantages of melatonin on seminiferous tubules of aged mice testes.

## Introduction

The pineal gland is the major source of the hormone melatonin (1). The importance of the pineal gland was noted long ago when, in the 16th century, it was thought to be the seat of the soul! However, only in the past three decades that remarkable advances in the knowledge of the functional significance of the gland’s hormone, melatonin, have been made. Nevertheless, correlation of endocrine activity and physiological role of melatonin is not entirely understood. For this reason, melatonin is attracting the interest of researchers (2). In 1954, Kitay and Altchule have demonstrated the influence of the pineal gland influences on reproductive function, thus establishing a link between the two (3). Melatonin receptors have been identified in hypothalamic neurons governing the release of pituitary gonadotrophs (4, 5), in gonadotropins of the anterior pituitary (6) and in both female and male gonads (7). Thus through the hypothalamic suprachiasmatic nucleus, melatonin influences the synthesis and release of GnRH and gonadotropin hormones. Nevertheless, the release of newly synthesized melatonin from the pineal gland into the circulation reaches the testes (8) and age-related reproductive decline is accompanied by progressive impairment of the neuroendocrine mechanisms that regulate leuteinizing hormone (LH) secretion. The biosynthetic activity of the pineal gland is markedly depressed and secretion of melatonin decreases significantly (9).

The aim of the present study is to evaluate whether the administration of melatonin in aged mice could influence the age-related changes in testes.

## Materials and Methods

Twenty male white mice, aged 16 months and 20-23g in weight were purchased from Iran Pasteur Institute and housed in a temperature-controlled room at 23 ± 2℃. Animals had access to food and water. They were cared for in accordance with the Principals and Guidelines of the Research Center at Tehran University of Medical Sciences. Mice were randomly divided into control and experimental groups with 10 animals per group. The experimental group received intraperitoneal injections of a daily single dose of 10 mg/kg melatonin (Sigma Chemical Co., USA) dissolved in saline for 14 days. The control group received only saline. Six days after the last injection all mice were sacrificed under ether anesthesia and the testes were excised, cut into small pieces and fixed in 10% formalin. Next, testes were dehydrated in an alcohol series, cleared in xylene, infiltrated with paraffin and embedded in paraffin. Paraffin blocks were cut into 5 µ thick sections and stained with hematoxylin and eosin. About 30 sections of seminiferous tubules that were round or nearly round were chosen randomly and measured for each group. The tubular diameter and height of the seminiferous tubule epithelium was measured at ×200 and ×400 magnifications using image analyzer Leica (DMLB) and Leica Qwin software. The diameter of the seminiferous tubule was measured across the minor and major axes, and the mean diameter obtained. We measured testicular interstitial and epithelium by the point counting method (10, 11). Testis tubules were evaluated for their modified spermatogenesis index (SI) by Johnson’s score (12). In Johnson’s score a grade from 1 to 10 was given to each tubule cross section according to the range from no cells to complete spermatogenesis.

### Statistical analysis

All tubular parameters were analyzed by SPSS software and student's t-test to assess the significance of changes between control and experimental groups.

## Results

**Fig 1:**
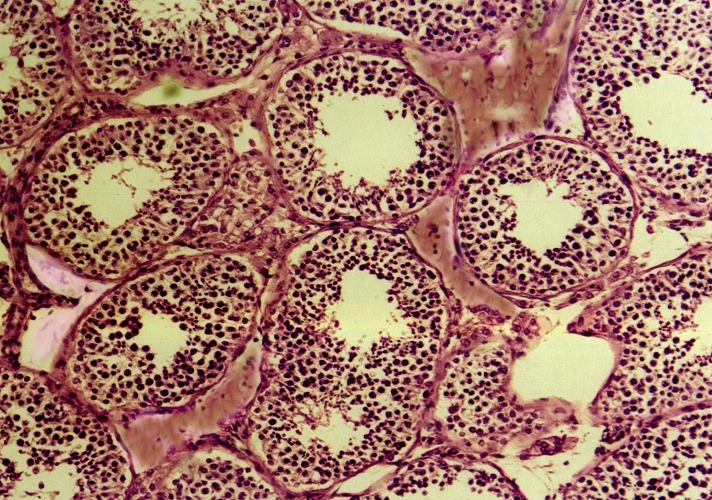
Photomicrograph of seminiferous tubules in the control group. The tubules are lined by germinal epithelium and surrounded by interstitial connective tissue (×200, H&E staining).

**Fig 2:**
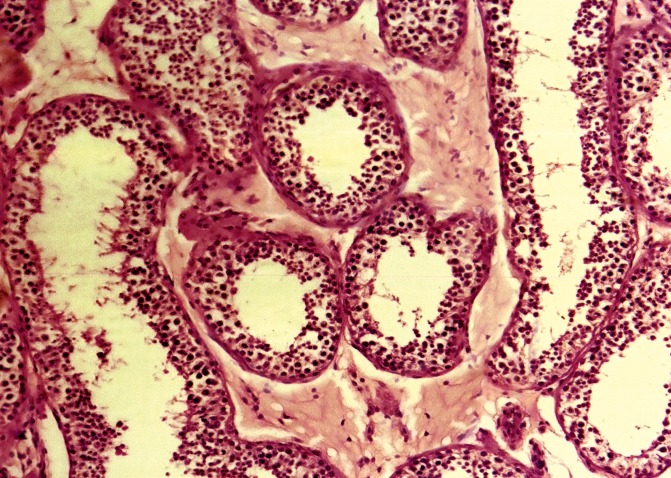
Photomicrograph of seminiferous tubules with wide lumen lined by low height germinal epithelium in the experimental group. The thick interstitial connective tissue is seen around the tubules (×200, H&E staining).

In the control group, the testes consisted of a number of seminiferous tubules lined by the germinal epithelium and separated by interstitial connective tissue (Fig 1). In melatonin-treated mice, the seminiferous tubules had a wide lumen lined by low height germinal epithelium and thick interstitial connective tissue (Fig 2).

**Fig 3:**
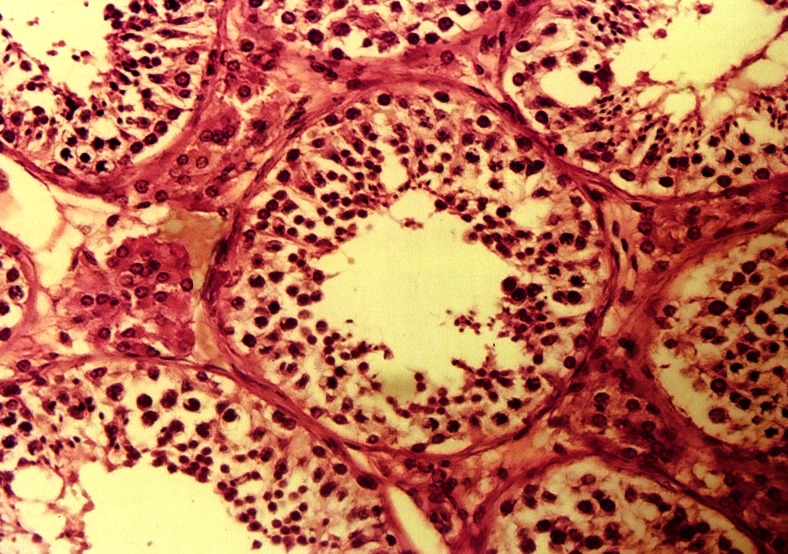
Photomicrograph of seminiferous tubules lined by cell series of germinal epithelium in the control group (×400, H&E staining).

**Fig 4:**
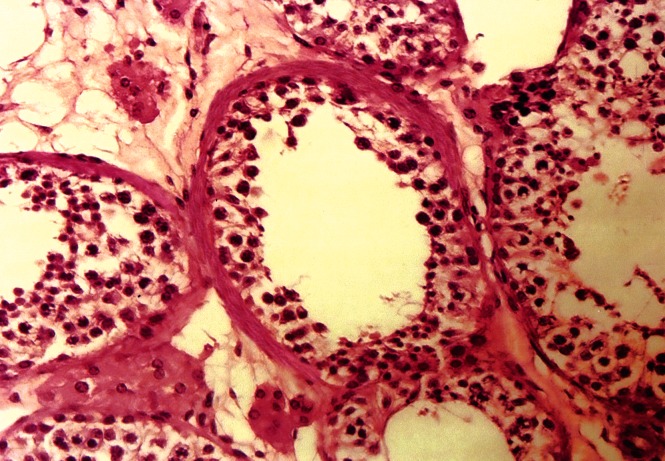
Photomicrograph of low germinal epithelium in seminiferous tubule of the experimental group (×400, H&E staining).

The germinal epithelium of tubules in the control group had cell series and few spermatozoa (Fig 3) whereas there was a reduction in the cells in the experimental group (Fig 4).

In morphometric measurements, the interstitial connective tissue had significant thickening in the experimental group (p<0.05). On the other hand, tubular diameter and germinal epithelium height decreased significantly (p<0.01). In addition, a reduction in the spermatogenic index was significant in the experimental group (p<0.001). Therefore the SI shifted from a level of 8 (few spermatozoa) to 5.5 (no spermatozoa and many spermatocytes) during the treatment with melatonin (Table 1).

**Table 1:** Values of morphological parameters obtained from seminiferous tubules of control and experimental mice


Analyzed parameters	Control group (Mean± SD)	Experimental group (Mean± SD)

Tubular diameter (micron)	174.3 ± 11.4	160 ± 10.9^*^
Germinal epithelium height (micron)	82.15 ± 6.3	76.8 ± 6.2^*^
Thickness of interstitial connective tissue (micron)	6.71 ± 2.25	18.98 ± 3.77^***^
Spermatogenesis Index (Score)	8 ± 2.64	5.5 ± 2.26^**^


n=30 sections for each group, *significant differences (p<0.01), **significant differences (p<0.001), ***significant differences (p<0.05).

## Discussion

In the present study, administration of melatonin to aged mice produced a marked decrease in tubular parameters in testes which confirmed earlier studies. The chronic administration of pineal extracts or pineal substances has the opposite effect from pinealectomy and restricts gonadal development or growth (13). Subcutaneous injection of melatonin into adult male hamsters caused involution of the testes (14) and intraperitoneal administration of melatonin to mice induced appearance of necrotic cells in seminiferous tubules and an increased incidence of aspermic tubules (15). Melatonin inhibited gonadotrophin-stimulated androgen production in hamster testes (8). In mice treated with melatonin the weight of testes decreased (16), the leydig cell nuclear volume and testosterone level also decreased (17, 18).

 Administration of melatonin and 5 methoxy tryptophane to mice caused a reduction in the diameter of seminiferous tubules (19). The unknown signaling pathway associated with the effect of melatonin on testicular activity involved down regulation of steroidogenic enzyme expression that lead to inhibition of androgen production (7). Although reduction in reproductive parameters have been associated with the exposure of melatonin, however there are reports of testicular growth in the animals with application of exogenous melatonin such that acceleration of testicular development was enhanced in juvenile animals (20). These observations are in agreement with correlations between the age of the animal and the effects of melatonin. Inactive adult hamster testes release more 5α-reduced compounds than active hamster testes. Androstane-3α 17βdiol is the main androgen produced from regressed testes under *in vitro* conditions (21). Employing semiquantitative RT-PCR has shown that melatonin reduces the expression of important steroidogenic enzymes (18). The observation that the sperm characteristics declined in the treatment group that received the higher dose of melatonin suggested that a dose as high as 9 mg could produce a suppressive effect on the hypothalamus. Consequently, gonadotrophin and testosterone hormones decreased which in turn the process of spermatogenesis reduced (20). Studies show that intra-testicular injection of melatonin decreases steroidogenesis by enhancing the primary effect of melatonin on leydig cell endocrine function along with reduced circulatory testosterone production and impairment of spermatogenesis (22). As with other organs, the reproductive system is subject to damage with toxic agents and radiation given the contribution of free radicals and related reactants to molecular and cellular destruction. Cellular damage is associated with increased malondialdehyde, myloperoxidase and decreased glutathione levels (23). Many investigators have studied the ability of melatonin as a radical scavenger to attenuate tissue injury. Similar studies on reproductive organs have confirmed the benefits of melatonin in resisting oxidative stress (24-26). Different opinions exist in the literature about the protective effects of melatonin against injuries, so that administration of melatonin after animal exposure to radiation did not have a significant effect, however the effect of melatonin did cause a significant reduction in micronuclei polychromatic erythrocytes in the bone marrow compared to controls. This suggests a cytotoxic effect of melatonin at a dose of 10 mg/ kg body weight (27). Hussein et al. have reported protective effects of melatonin against X-ray induced acute testis damage in rats but they suggested that the clinical ramification of their observations mandate further studies (28). Sonmez et al. showed the protective effect of melatonin with vitamin E on antioxidant enzyme activities and sperm characteristics of homocysteine-treated male rats at a 1 mg/ kg dose of melatonin (29). The dose and the duration of melatonin treatment are thought to be important points. In fact, melatonin at a low dose and for less than one week in studies have shown a protective role in injured tissues. Melatonin mediates the gonadal hormone secretion and at the higher dose of 9 mg/kg has an adverse effect on the process of spermatogenesis (18, 20, 22). As the present results indicates that melatonin at a dose of 10 mg/kg for 14 days exerts inhibitory action on seminiferous tubules in aged mice.

 Another finding of this research was thickening of interstitial connective tissue of the seminiferous tubules. The literature has described interactions between extracelluar matrix, tubular wall and germinative cells as important to their normal development (30). Some studies have demonstrated an important increase in the amount of collagen resulting in interstitial fibrosis and a thickened lamina propria of the seminiferous tubules which prevents the development of germinative cells. The testicular interstitium and lamina propria of patients treated with gonadotrophins have shown significantly less collagen system fibers (31).

## Conclusion

The results of this study showed the disadvantages of melatonin on seminiferous tubule parameters of aged mice.
